# Key factors influencing post-diagnostic support and care planning for people with dementia from South Asian backgrounds: a systematic review of qualitative studies

**DOI:** 10.1186/s12877-026-07064-y

**Published:** 2026-01-31

**Authors:** Pushpa Nair, Emily Spencer, Kate Walters, Fiona Stevenson, Jemima Dooley, Nathan Davies

**Affiliations:** 1 Department of Primary Care and Population Health, University College London, London, UK; 2 Institute of Epidemiology and Healthcare, University College London, London, UK; 3 Mood Disorders Centre, University of Exeter, Exeter, UK; 4 Wolfson Institute of Population Health, Queen Mary University of London, London, UK

**Keywords:** Inequalities, Ethnic minorities, Dementia, Culture, Ageing

## Abstract

**Background:**

Dementia in minority ethnic groups is on the rise. South Asian people represent 9.3% of the UK population and are the largest minority ethnic (ME) group. Most dementia care in South Asian communities is provided at home by family carers, yet there is low uptake of care planning and post-diagnostic support services. This review explored factors influencing care planning and post-diagnostic support for South Asian people with dementia.

**Methods:**

Systematic review and thematic synthesis of qualitative studies exploring care planning and post-diagnostic support experiences and views of South Asian people with dementia, family carers, and health and social care professionals (HSCPs). There were no limits on country or date of publication. Six databases were searched (inception - June 2024) and two reviewers independently screened and quality-appraised studies. Reflexive thematic analysis was used to generate themes, which were then mapped to the Socioecological Model. The review protocol was registered on PROSPERO [Registration number: CRD42023404125].

**Results:**

From 3165 studies found, 2069 were screened (after de-duplication) and 24 studies were included for thematic synthesis. Key influential factors were mapped to one of four levels of a modified version of the Socio-ecological Model (SEM): (1) *Individual and community level factors* - cultural duty for home care, stigma and misinformation, support networks; (2) *Interpersonal level factors* - language barriers and person- and family-centred care; (3) *Organisational and systems level factors* - lack of integrated support, cultural competence, system pressures and institutional discrimination; (4) *Structural level factors* - socioeconomic and policy considerations.

**Conclusions:**

Recommendations to improve dementia care planning and post-diagnostic support for South Asian communities include (1) Reframing narratives around dementia and help-seeking through culturally-tailored community interventions; (2) Culturally competent, person- and family-centred care; (3) Holistic and integrated support beyond clinical care; (4) Equitable partnership working with South Asian communities to co-produce dementia services.

**Supplementary Information:**

The online version contains supplementary material available at 10.1186/s12877-026-07064-y.

## Background

Dementia is a pressing global concern, with over 55 million people affected. This number is predicted to rise to 139 million by 2050 – with much of the increase to be seen in developing countries and ethnic minorities [1]. In the UK (United Kingdom), the number of minority ethnic (ME) people with dementia is projected to rise sevenfold by 2051, compared to just twofold in the general population, reflecting a greater migrant ageing population, higher rates of young onset dementia in these groups, and greater risk factors for vascular dementia [2, 3]. ME groups also experience marked inequalities in dementia care, including delayed diagnosis, reduced service access [4, 5] and disparities in prescribing [6, 7].

South Asian people remain underrepresented in both dementia research [8] and service usage [4, 9]. There is often low awareness and poor understanding of dementia as a medical condition, with greater stigma in South Asian communities [4, 9]. UK-based studies suggest that most South Asian older adults with dementia are cared for at home, influenced by cultural expectations, stigma associated with social care use, and fears over culturally-insensitive care [4, 9], leading to greater help-seeking at crisis point [10]. Health and social care professionals (HSCPs) may assume South Asian communities ‘look after their own’ [11] and do not require external help [12], which influences service design and delivery [13]. Evidence shows a growing desire for help among the South Asian community, yet existing dementia diagnostic pathways and post-diagnostic services are rarely culturally inclusive or tailored to their needs [9, 14–16].

Effective post-diagnostic support and care planning might address some of the inequalities in care. Post-diagnostic support covers a wide range of services and information for individuals with dementia and carers after diagnosis (for example, social care, financial support, dementia services and information, support groups) and incorporates care planning as a key element [17]. Whilst there is a large degree of overlap between what constitutes post-diagnostic care and care planning, care planning is a more specific iterative process, whereby HSCPs have discussions with patients/care recipients to outline care preferences, health and wellbeing goals and support needs (for them and their carers), ideally in the form of a written document [18, 19], supporting shared decision-making. The remit of care planning includes dementia reviews, written care plans, care coordination, individualised information and service signposting, risk assessments, medication reviews, eliciting cultural and religious care preferences, and advance care planning (ACP) [20]. NHS England [20] recommends a care plan for every person with dementia, but there is evidence that this process is underused and of variable quality and/or benefit for people with dementia [21, 22]. Research on dementia care planning for ME groups is lacking. Most studies have focused on ACP (which outlines preferences for future care) and report overall improved outcomes for people with dementia [23, 24], although ACP rates amongst ethnic minorities are low [25], with more documented barriers (such as language, cultural factors and mistrust) [26, 27]. Previous reviews have explored pathways to diagnosis, South Asian cultural attitudes towards dementia and caring, and inequalities in dementia care [4, 9, 15, 16, 28]. This review therefore seeks to focus on post-diagnostic support and care planning, which have been much less documented.

Given the limited literature on formal care planning and post-diagnostic support for South Asian people with dementia, the aim of this review was to (a) explore factors influencing service use, interactions and engagement with health and social care bodies, and (b) consider how care planning and post-diagnostic support interventions might be designed and adapted to meet the needs of people with dementia/their family carers from South Asian backgrounds.

## Methods

A systematic review and thematic synthesis of qualitative studies [29] was conducted according to the Enhancing Transparency in Reporting the Synthesis of Qualitative research (ENTREQ) guidelines [30], in an approach to identify key practice recommendations. The review protocol was registered on PROSPERO [Registration number: CRD42023404125].

### Searches

We searched Ovid MEDLINE, EMBASE, PsychINFO, CINAHL, Web of Science, and ASSIA from inception to 19th June 2024 (last search date across all databases). The search strategy was developed in conjunction with an information specialist from the university library, using Medical Subject Headings (MeSH) and keywords, trialled on Medline, and adapted to each database. Given that care planning is difficult to define in search terms, we included all qualitative studies on dementia in South Asian populations for initial title/abstract screening. Search terms focused on Dementia*, South Asia*, and Qualitative* (see Supplementary Appendix 1, Additional File 1 for the Medline search strategy, which is representative for other database searches). Additionally, citation index tracking using Scopus and hand-searching reference lists of shortlisted full-text papers and prior systematic reviews were conducted.

### Eligibility

This review included studies with a focus on any aspect of care planning or post-diagnostic support in dementia that incorporated the views and/or experiences of South Asian people with dementia ≥60 years old, family carers or HSCPs. Inclusion and exclusion criteria are provided in Table 1 below.

**Table 1:** Systematic review inclusion and exclusion criteria

	Inclusion	Exclusion
Population	South Asian older adults aged >60 years, with a diagnosis of dementia;Family carers of South Asian people with dementia; Health and social care professionals (HSCPs) caring for South Asian people with dementia	Studies with mixed participants where < 50% of participants met the inclusion criteria (and findings not reported separately for each group); Paid carers; Family carers aged <18 years
Phenomenon of interest	Dementia care planning, including Advance care planning (ACP); Dementia post-diagnostic support	Focus on care prior to dementia diagnosis; Focus solely on family caregiver burden or general attitudes to dementia.
Context	Any relevant health, community or social care setting (primary care, community services, residential/nursing care, secondary care)	No limits on country
Study type	Qualitative studies; Mixed methods, where qualitative findings are reported separately; Primary and secondary analyses	Quantitative studies; Surveys; Literature reviews; Conference abstracts; Commentaries; Study protocols; Book chapters; Grey literature; Theses

### Study selection

All identified studies were uploaded to EndNote for de-duplication and then screened in Rayyan [31]. The primary reviewer (PN) assessed all titles and abstracts against the inclusion/exclusion criteria, with 20% screened independently by a second reviewer (ES) achieving 96% concordance. All full texts were screened by PN and 20% screened by ES, with 97% agreement. Discrepancies were resolved through wider team discussion.

### Data extraction and synthesis

Data were extracted using a custom template (see Supplementary Appendix 2, Additional File 2), covering study aims, setting, sample, data collection, analysis and main findings. Quality was assessed using the Critical Appraisal Skills Programme (CASP) Qualitative Studies Checklist [32]. Most studies were of good or reasonable quality. Whilst no studies were excluded on the basis of quality assessment alone [33], studies of low quality were not given as much weighting in the analysis (for example, key themes were not extrapolated from weaker studies). Quality assessment and data extraction was led by PN, with ES independently checking 10% of studies.

**Fig. 1:**
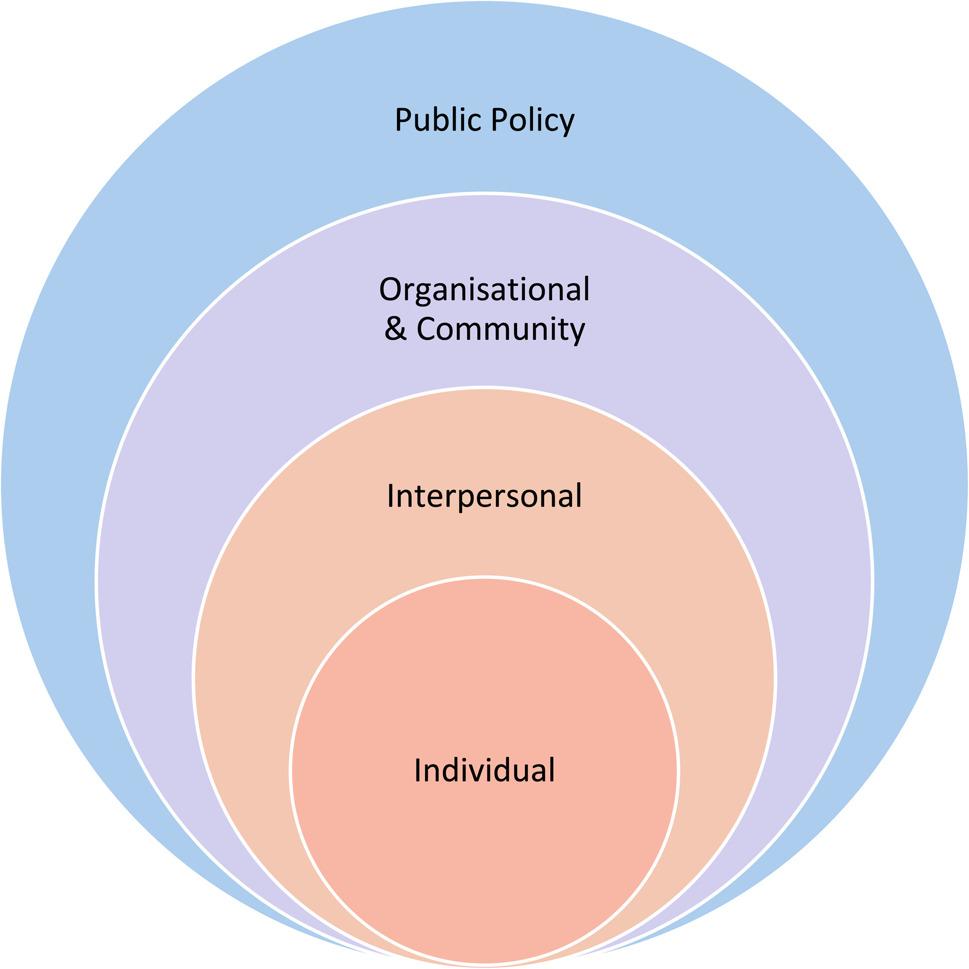
Modified Socio-ecological model (SEM) (derived from McLeroy et al. 1988 [37])

For thematic synthesis, a reflexive thematic analysis approach [34] was used, which forefronts the researcher’s unique positionality as the primary instrument of data analysis. PN is an academic General Practitioner (GP) from a South Asian background. PN utilised a reflexive approach to critically engage with the data and actively develop codes and themes. A reflexive thematic analysis approach does not require second coder checks for trustworthiness. PN inductively coded text from the findings and discussion sections of studies using NVivo 14 software [35]. Codes were used to generate initial descriptive themes. Analytical themes were then developed to ‘go beyond’ the original data [29] and mapped to a modified version of the Socio-ecological Model (SEM) [36, 37]. The original SEM (Bronfenbrenner, 1979 [36]), highlights the dynamic interplay between individuals and their environment on multiple levels and how this influence behaviours and attitudes. The modified SEM (McLeroy et al. 1988 [37]) has been widely used in healthcare research as a way of understanding contextual factors influencing help-seeking behaviours, as well as barriers and facilitators to accessing support. Figure 1 illustrates the levels of the modified SEM [37]: Individual, interpersonal, organisational and community, and public policy. Whilst coding was done primarily by PN, themes were developed, refined and reviewed with all authors to enhance rigor and reach consensus. Demographic factors like ethnicity, socioeconomic status and gender were also compared within and across themes.

## Results

**Fig. 2:**
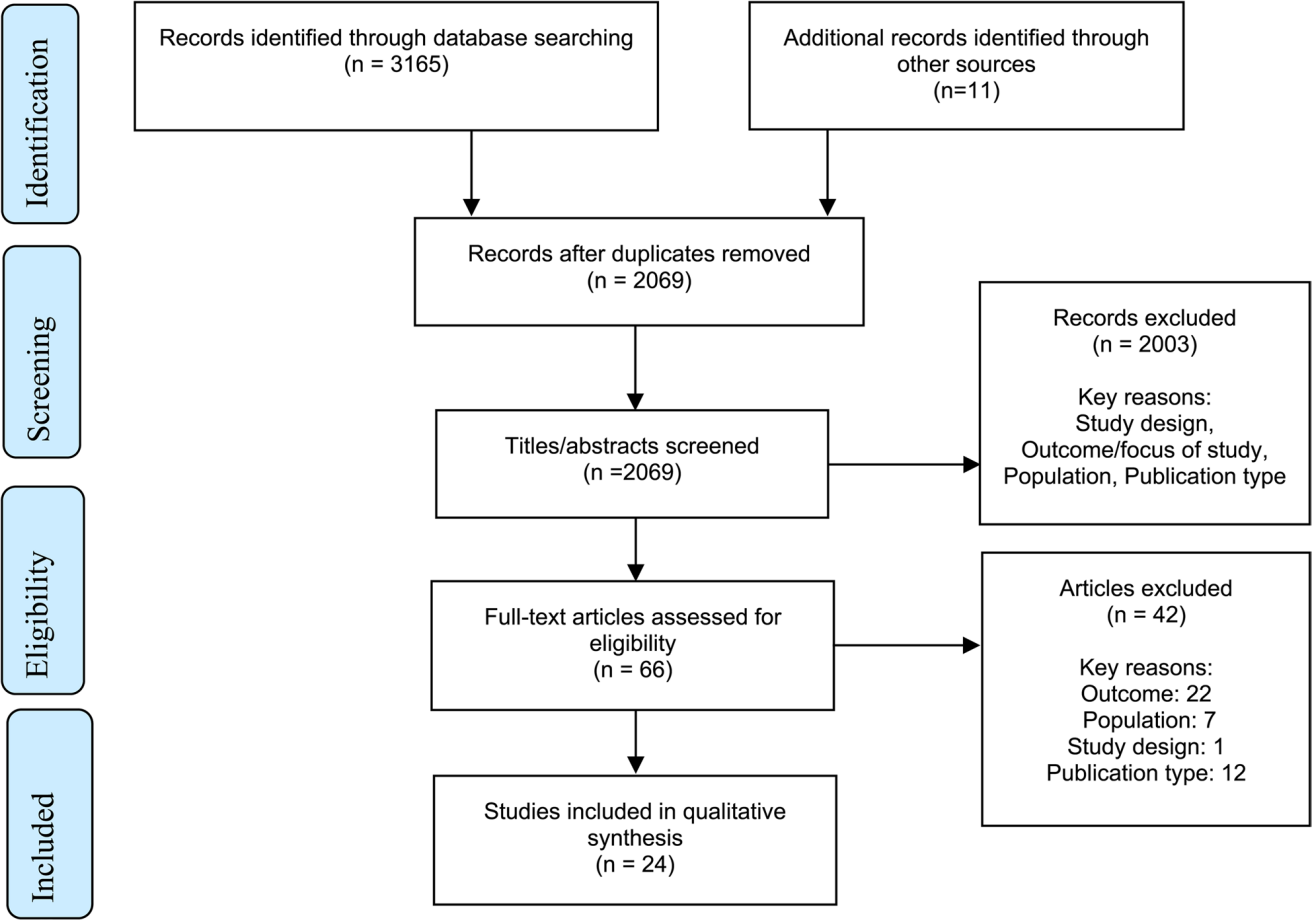
PRISMA diagram showing study selection process

Database searches identified 3165 articles, and 11 additional papers were retrieved from citation tracking and hand searches. After de-duplication, 2069 citations were title/abstract screened, with 66 articles retrieved for full text screening. 24 eligible articles were included in the thematic synthesis. The screening process is summarised in the PRISMA flowchart (Fig. 2).

### Study characteristics

83% of included studies (See Table 2) were from higher-income countries where South Asians are a minority population - the UK (*n* = 17), New Zealand (*n* = 1) and Australia (*n* = 2). Four studies reported on lower-income countries where South Asian are a majority population - India (*n* = 3) and Pakistan (*n* = 1). Publications spanned 2003 to 2024.

**Table 2:** Characteristics of included studies

Study	Aims	Participants & Setting	Post-diagnostic support elements explored in paper	Care planning elements explored in paper	Methods
*Countries where South Asians are a minority population*					
Armstrong et al. 2022 [38] UK (London) *Study ID: S1*	Explore the impact of Covid-19 on the care of Black and South‐Asian people with dementia and carers.	*n* = 8 [6 Family carers (90% > 50 years old); 2 People with dementia (> 65, age range 76–88; M) -Indian, Pakistani] *Setting: Community/Home*	Signposting to services; Social care	Communication with HSCPs; Family involvement in decision-making; Person-centred care	Semi-structured qualitative interviews **Analysis**: Thematic analysis
Baghirathan et al. 2018 [39] UK (Bristol) *Study ID: S2*	To explore the experiences of caregivers from 3 different ethnic minority groups (South Asian, Black Caribbean and Chinese backgrounds) of dementia care.	*n* = 47 [7 Family carers (2 F; 5 M; Hindu, Muslim, Sikh); 40 Professionals (South Asian-led Voluntary and Community Sector Organisations, VCSOs)] *Setting: Community/Home*	VSCOs; South Asian specific services; Statutory services (e.g., Day centres)		Semi-structured qualitative interviews;Focus groups **Analysis**: Grounded Theory
Bowes & Wilkinson, 2003 [40] UK (Scotland - Glasgow, Edinburgh) *Study ID: S3*	To explore (1) experiences of South Asian people with dementia and family carers; (2) service support; 3) key issues for service professionals	*n* = 11 + case studies [4 People with dementia and their family carers (Indian, Pakistani); 11 HSCPs (clinicians, VCSOs)] *Setting: Community/Home*	VSCOs; South Asian specific services; Statutory services	Carer support; Information provision	Semi-structured qualitative interviews (HSCPs); Case studies (carers and people with dementia) **Analysis**: Thematic analysis
Brijnath et al. 2021 [41] Australia (Melbourne, Sydney, Perth) *Study ID: S4*	To describe ethnic minority families’ pathways to a dementia diagnosis and accessing support services using the concept of sense-making.	*n* = 37 [18 Family carers (Indian); 19 Professionals (service-related)] *Setting: Community/Home*	Post-diagnostic support pathways	Information provision	Semi-structured narrative video interviews **Analysis: T**hematic analysis – storyboards to co-produce films; Narrative analysis − 3 case studies
Du Toit et al. 2023 [42] Australia (Sydney) *Study ID: S5*	To explore (within Sydney-based residential aged care facilities): (1) effectiveness of current culturally appropriate dementia care practices; (2) barriers for providing culturally appropriate dementia care.	*n* = 16 [8 Family carers (71% > 60 years old; 6 F, 2 M; Indian); 8 Professionals (Care home staff)] *Setting: Care home*	Care homes; Cultural competence in services	Cultural and religious care preferences recorded in care plans; Family involvement in care plans; Information provision	Nominal group technique (NGT) groups ***Analysis***: Thematic analysis on consensus statements generated by each NGT groups
Forbat, 2004 [43] UK (S. England, Midlands) *Study ID: S6*	To explore structural inequalities as a form of ‘institutional’ abuse of carers and care recipients, focusing on racism and institutional discrimination.	Family carers (n = not stated) *Setting: Community/Home; in-patient*	Cultural competence in services		Qualitative narrative interviews **Analysis**: Thematic analysis; Discourse analysis; resulting in 2 South Asian case studies
Herat-Gunaratne et al., 2020 [44] UK (London, Bradford) *Study ID: S7*	To explore how carers’ cultural backgrounds influenced their experiences, negotiation of the caring role and relationship with services.	*n* = 10 [Family carers (mean age 49.4, range 32–69; 5 F, 5 M; Indian, Bangladeshi)] *Setting: Community/Home*	Social care (care workers)	Preferred place of care; Cultural and religious care preferences	Semi-structured qualitative interviews **Analysis: T**hematic analysis
Hossain, 2021 [45] UK *Study ID: S8*	To highlight the impact of dementia on Bangladeshi caregivers and help improve the advice, care, and support available for other ethnic minority caregivers.	*n* = 15 [Family carers (Bangladeshi; 12 F, 3 M)] *Setting: Community/Home*	Social services (care workers, financial); Carer support	Cultural and religious care preferences	Semi-structured qualitative interviews **Analysis**: Thematic analysis
Hossain et al. 2022 [46] UK (W. Midlands) *Study ID: S9*	Explore perspectives of carers from point of diagnosis to end of life.	*n* = 16 [Family carers (age range 20–80; M 9, F 7; Indian, Bangladeshi, Pakistani)] *Setting: Community/Home; Secondary Care*	Social care (care workers)	Advanced care planning and end-of life care; Cultural and religious care preferences	In-depth, semi-structured qualitative interviews **Analysis**: Thematic analysis
Hossain & Khan, 2020 [47] UK (London, Portsmouth) *Study ID: S10*	Examine the barriers to dementia health care service use in the Bangladeshi community.	*n* = 6 [Family carers – Bangladeshi] *Setting: Community/Home*	Information signposting; Social care; South Asian specific services; S ervice access barriers	Preferred place of care; Cultural and religious care preferences	Semi-structured qualitative interviews **Analysis**: Thematic analysis
Hussain, Clark & Innes 2024 [48] UK *Study ID: S11*	To explore cultural understandings and experiences related to dementia, stigma and using dementia services in the Bangladeshi community.	*n* = 26 [10 Family carers (age range 30–65); 10 People with dementia (70% >65 years old; 90% >60) - Bangladeshi; 10 Professionals (service providers)] *Setting: Community/Home*	Cultural attitudes to support services		Semi-structured qualitative interviews **Analysis**: Thematic analysis
James et al. 2023 [49] UK (London, Leicester) *Study ID: S12*	To explore the care and support received and wanted by UK South Asian and White British people affected by dementia and whether access to care is equitable.	*n* = 42 [11 Family carers (mean age 54.5, range 22–87; 7 F, 4 M); 6 People with dementia (mean age 75.5, range 65–86; 5 F, 1 M) - Bangladeshi, Pakistani, Sri Lankan and Indian; 25 Professionals (clinicians)] *Setting: Community/Home*	Service access barriers; Cultural competence in services	Cultural and religious care preferences	Semi-structured qualitative interviews **Analysis**: Thematic analysis
Jenkins & Kamala, 2024 [50] UK *Study ID: S13*	Understand nurses’ experiences of providing support to South Asian people with dementia and their families, and to identify barriers and enablers of good transcultural care in in-patient and community settings	*n* = 15 [Professionals - nurses] *Setting: Community; in-patient*	Use of interpreters	Communication with HSCPs; Cultural and religious care preferences; Family involvement in care plans; Person-centred care	Semi-structured qualitative interviews **Analysis**: Thematic analysis
Jutlla, 2015 [51] UK (Wolverhampton) *Study ID: S14*	To explore how migration experiences and life histories impact on perceptions and experiences of caring for a family member with dementia for Sikhs living in Wolverhampton.	*n* = 12 [Family carers (Sikh; age range 44–83; 9 F, 3 M)] *Setting: Community/Home*	Service access barriers; Cultural attitudes to support services		Narrative qualitative interviews **Analysis**: Grounded theory (data presented in the form of 3 case studies)
Jutlla & Arblaster, 2023 [52] UK (London, Midlands) *Study ID: S15*	To understand experiences of post-diagnostic dementia support for the South Asian community in England to identify whether their support needs are being met and what their needs are.	*n* = 13 [12 Family carers (age range whole sample 28–78; 8 F, 4 M); 1 Person with dementia (78 years old, F) - Sikh, Muslim and Hindu backgrounds] *Setting: Community/Home*	Service access barriers; Cultural competence in services; Information provision/signposting; Post-diagnostic support pathways	Cultural and religious care preferences	Qualitative semi-structured interviews/conversations **Analysis**: Thematic analysis (3 case studies derived from analysis)
Krishnamurthi et al. 2022 [53] New Zealand (Auckland) *Study ID: S16*	To explore how dementia is perceived, experienced, and managed from the perspective of Indian people with dementia in Aotearoa, New Zealand and their family caregivers.	*n* = 15 [10 Family carers (median age 61, range 41–81; 6 F, 4 M); 5 People with dementia (median age 74, range 65–77; 1 F, 4 M) – Indian] *Setting: Community/Home*	Information provision/signposting; Post-diagnostic support pathways; Home care (care workers); Day centres	Cultural and religious care preferences; Preferred place of care	Semi-structured qualitative interviews **Analysis**: Thematic analysis
Lawrence et al. 2008 UK (London) [54] *Study ID: S17*	To explore the caregiving attitudes, experiences and needs of family carers of people with dementia from the three largest ethnic groups in the UK (South Asian, Black Caribbean, White British).	*n* = 10 [Family carers (age range of whole sample 33–87; 5 F, 5 M; South Asian) *Setting: Community/Home*	Service access barriers; Cultural attitudes to support		In-depth qualitative interviews **Analysis**: Grounded theory
Lawrence et al. 2011 [55] UK (London) *Study ID: S18*	To explore the reality of living with dementia from the perspective of people with dementia within the three largest ethnic groups in the UK (South Asian, Black Caribbean, White British).	*n* = 9 [People with dementia (mean age 77, range 65–87; 5 M, 4 F – Indian and E. African backgrounds)] *Setting: Community/Home*	Cultural attitudes to support		In-depth qualitative interviews and vignettes. **Analysis**: Grounded theory
Nair et al. 2022 [56] UK (London) *Study ID: S19*	To explore the meaning of food, the impact of dementia on eating and drinking, and carers’ experiences of support in minority ethnic groups.	*n* = 10 [7 Family carers (mean age 55.6, range 38–81); 3 People with dementia (mean age 73.3) - Indian, Pakistani, Bangladeshi] *Setting: Community/Home*	Cultural competence in services; Cultural attitudes to support	Cultural and religious care preferences (food)	Semi-structured qualitative interviews **Analysis**: Thematic analysis
Parveen, Blakey and Oyebode, 2018 [57] UK *Study ID: S20*	Evaluate impact/effectiveness of an Information Programme for South Asian families (IPSAF) who support an individual living with dementia	*n* = 37 [34 Family carers; 3 People with dementia - Indian, Pakistani, Bangladeshi] *Setting: Community/Home*	Culturally adapted carers’ information programme		Intervention evaluation via focus groups and semi-structured interviews **Analysis**: Thematic analysis
*Countries where South Asians are a majority population*					
Balouch et al. 2021 [58] Pakistan (Karachi, Lahore) *Study ID: S21*	To explore (1) whether culture/religion plays a role in caregiving; (2) how family caregivers cope, barriers they face and what help is acceptable; (3) how findings can help raise awareness/influence public policies.	*n* = 20 [Family carers (age range 35–80; 14 F, 6 M; Pakistani] *Setting: Community/Home*	Day Centres; Home care (care workers); Cultural attitudes to support; Service access barriers; Lack of services	Information provision/signposting	Semi-structured qualitativeinterviews **Analysis**: Thematic analysis
Brijnath et al. 2024 [59] India (Bengaluru) *Study ID: S22*	Examine how autonomy, equality, dignity, and personhood are practiced in the care of people living with dementia at home in urban India, using the concept of relational solidarity	*n* = 44 [19 Family carers (mean age 50; 9 F, 10 M; Indian); 25 HCPs] *Setting: Community/Home*		Person-centred care	Videorecorded semi-structured qualitative interviews. Data derived from the *Moving Pictures India* project. **Analysis**: Thematic analysis
Hurzuk et al. 2022 [60] India (Delhi, Chennai) *Study ID: S23*	To understand the perceptions, attitudes and beliefs of dementia in India.	*n* = 43 [19 Family carers (age range 19–77; 13 F, 6 M); 8 People with dementia (age range 60–82; 88% >65 years old; 4 F, 4 M) -Indian; 16 HCPs] *Setting: Community/Home*	Dementia competency (HCPs); Service access barriers; Cultural attitudes to support		Focus groups Semi-structured qualitative interviews **Analysis**: Thematic analysis
Lamech et al. 2019 [61] India (Chennai) *Study ID: S24*	To explore the needs and challenges of family caregivers in Chennai, India.	*n* = 19 [Family carers (mean age 51; 11 F, 8 M; Indian Tamil)] *Setting: Community/Home*	Dementia competency (HCPs); Service access barriers		Focus groups; In-depth qualitative interviews **Analysis**: Thematic analysis

Most studies (*n* = 23) examined dementia care in community settings, with some also exploring secondary care (*n* = 3) or care homes (*n* = 1). Research methods included semi-structured interviews (*n* = 15), in-depth interviews (*n* = 3), narrative interviews (*n* = 3), focus groups (*n* = 7), case studies or vignettes (*n* = 2), with eight mixed methods studies. Most studies (*n* = 20) employed thematic analysis, with four using grounded theory.

Participants included South Asian people with dementia (mean age > 65 years old) and family carers from Indian (including Tamil and Sikh), Pakistani, and Bangladeshi backgrounds. Eight studies gathered views from people with dementia, 22 from family carers and nine from HSCPs (doctors, nurses, voluntary sector workers and statutory service providers). Most studies explored experiences with community-based post-diagnostic support and cultural influences on care planning; one examined ACP, and another explored communication and interactions with healthcare professionals (HCPs). Table  3 reports on the quality assessments for each study.

**Table 3:**
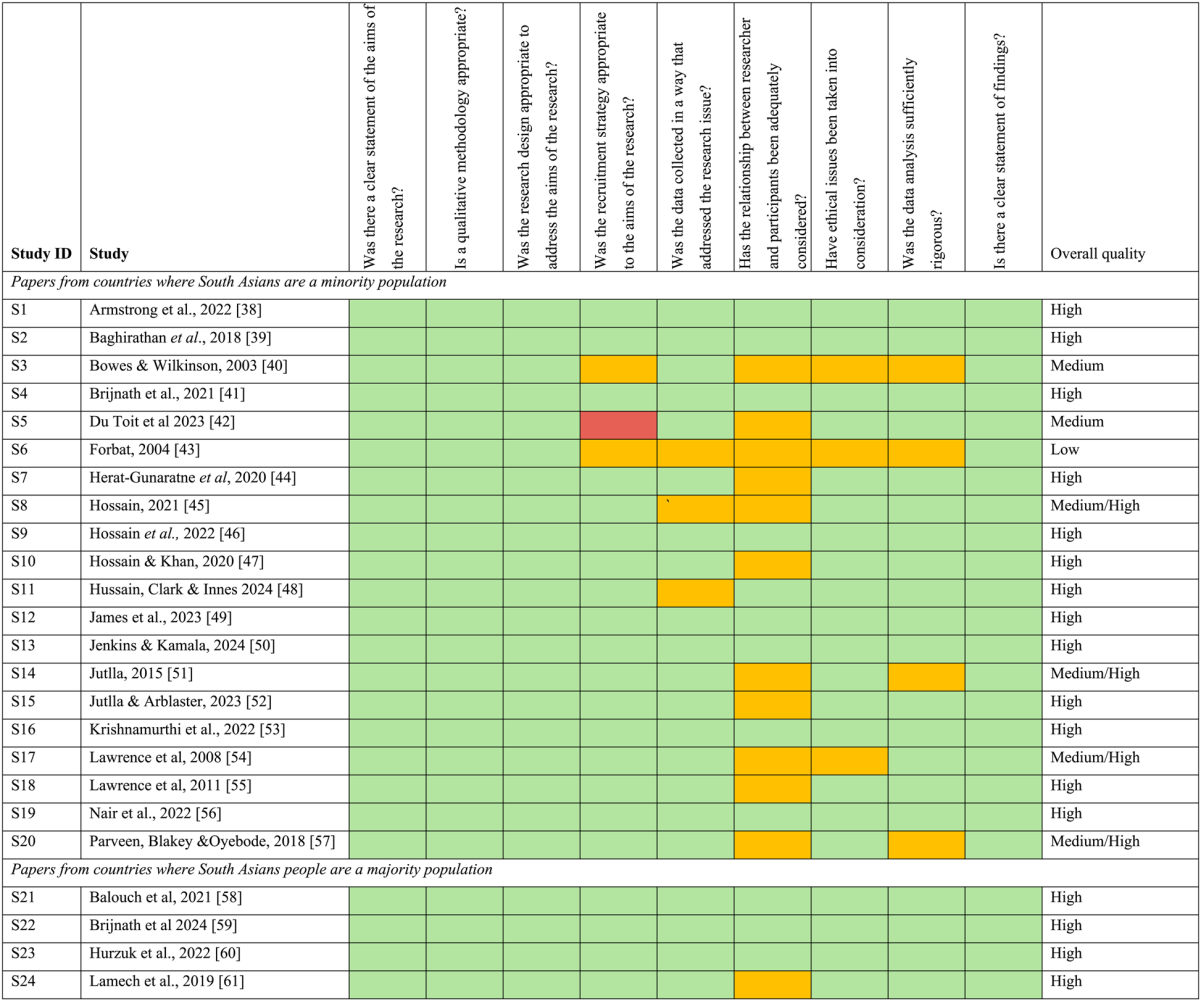
Quality appraisal summary of included studies [38–61]

Key:  = Yes  = Can't tell  =No

### Themes

**Fig. 3:**
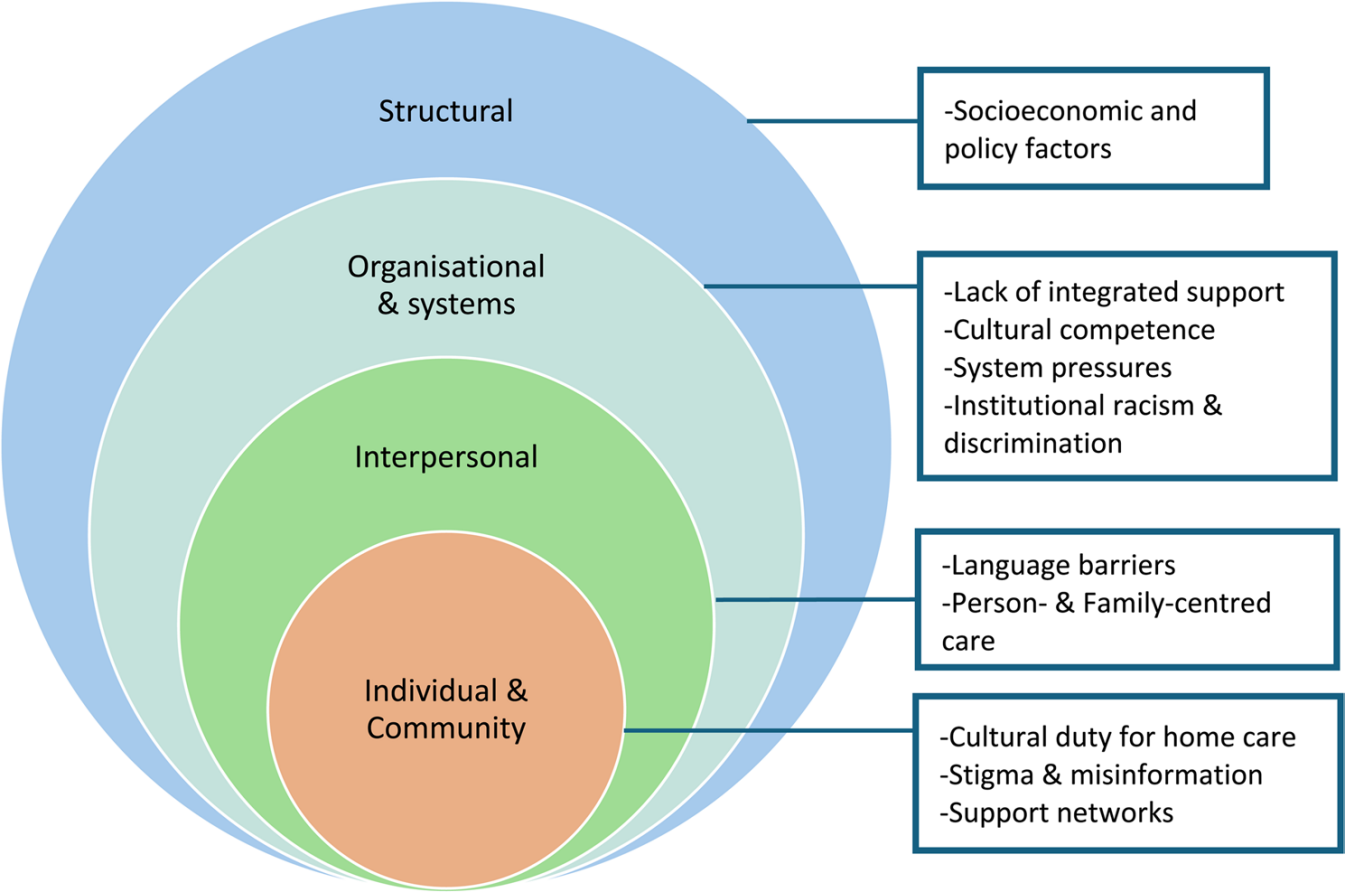
Adapted Socio-ecological Model (SEM) summarising influential factors on the dementia care planning process and uptake of post-diagnostic support services in South Asian communities

Analysis of the 24 papers included in this review identified several influential factors, barriers and facilitators that impacted attitudes, acceptability and access to post-diagnostic support services and engagement with care planning. In order to more accurately convey and map our findings, we adapted the modified version of the SEM [37] in the following ways: we aligned ‘Individual’ and ‘Community’ level factors together, to better illustrate the influential interplay between culture and the individual within South Asian communities; we also added ‘Systems’ to the ‘Organisational’ level, to capture interactions between different organisations and wider institutions; instead of ‘Public Policy’, we have included a broader ‘Structural’ level, to incorporate both public policy and socioeconomic factors. The levels of analysis for our adapted SEM are: 1.) individual and community; 2.) interpersonal; 3.) organisational and systems; 4.) structural. The SEM model is heavily weighted towards findings from higher-income, South Asian-minority countries, as these studies comprised 83% of the review; where differences between settings were noted in the analysis, these are reported within each theme below. Themes are summarised in Fig. 3.

### Individual and community level

#### Cultural duty for home care

All papers (from countries where South Asians represent both a minority and majority group) highlighted a strong cultural duty amongst South Asian families to care for relatives with dementia at home, linked to family (especially filial) responsibility, honour, or religious duty. However, for some, this was experienced as more of an obligation or community expectation, particularly from the older generation, leading to stigma, fear of judgement and reluctance to access support services, or non-acceptance of outside services by people with dementia themselves [44, 50, 51, 56, 58]. This contributed to increased carer burden, especially impacting female family carers due to gendered expectations around caregiving:

*But the South Asian community*,* when I consider my father-in-law*,* he always thinks that it’s his wife’s duty [to undertake all caring duties] and he didn’t need any carers [care workers] to come in and go out*,* it all has to be done by his wife. (Male former carer*,* son-in-law*,* UK* [56]*)*

Across all papers, there was a strong preference for services supporting home-based care. This included a desire for more care workers (to provide care for the person with dementia at home), information about managing advanced dementia at home (including incontinence and behavioural and psychological symptoms of dementia), respite, and psychosocial support for family carers, as well as help with more practical things, such as accessing financial benefits, advocacy and legal support.

*We don’t need advice anymore. We need somebody to come and help us. We need somebody to come and give us some respite. We need money. We need funding. We don’t need talks. (Carer*,* UK* [40]*)*

Only participants in one study [53] had direct experience of care homes, reflecting a low uptake amongst South Asian communities, with participants across several papers expressing negative attitudes:

*“Manush-e Kita Khoibo?” [what are people going to say? ]. How could we show our face to the community? It is not our culture to send older people to the care home. However*,* my children will also not feel good to send their mum to a care home. (Person with dementia*,* female*,* UK* [48]*)*

#### Stigma and misinformation

Most papers (from countries where South Asians represent both a minority and majority group), noted low awareness of dementia as a *medical* condition, particularly amongst the older generation, with many attributing symptoms to normal ageing, cultural isolation, stress or diet. Some framed dementia as a spiritual issue or equated it with mental illness, increasing stigma around the condition. This often led to reduced engagement with health and social care services, with some families choosing spiritual healers over medical help, leaving them vulnerable to exploitation. Embarrassment, stigma and shame around dementia resulted in social withdrawal and diagnoses being kept hidden within families to maintain social standing.

*I tell my friends and extended family members that I cannot remember things or forget recent events. However*,* I do not tell them that I have dementia; people will not understand what dementia is*,* and they may judge me. People are not educated. People might say- “e beti pagol oi gese” [she has gone mad]; then the whole community may gossip about my family and me*,* which may damage my family’s “izzat” [honour]. (Person with dementia*,* female*,* UK* [48]*)*

One paper [46] also highlighted stigma in the South Asian community around discussing death, future wishes and financial planning. Facilitators to overcome stigma and misinformation included rethinking terminology, developing culturally tailored education programs in partnership with community and religious organisations, and spreading awareness through local or national media platforms, such as TV, South Asian radio stations and social media:

*… like social media that informs that what it is*,* what kind of disease it is. I have read and learnt a lot of things relating to the disease of my mother-in-law from the Internet. My husband has also read about it. (Female carer*,* daughter-in-law*,* Pakistan* [58]*)*

#### Support networks

Across both higher- and lower-income settings, most South Asian people with dementia relied on informal, often multigenerational, family networks, sometimes supported by neighbours. This could influence how care needs were perceived by HSCPs, particularly in care settings:

*…it’s nice to see that they’ve all got a big family and it creates a positive impact on them I suppose*,* seeing familiar faces and making sure that their family are there every day and making sure their needs are met. Sometimes when they’re here we don’t really need to do anything for them…so*,* say when families do come they’ve actually said*,* oh no they like to bath them*,* they like to feed them*,* they like to take them to the toilet and they like to be involved in their care… (Professional – in-patient nurse*,* UK* [50]*)*

Informal support networks were often preferred over formal care for meeting cultural needs, as well as offering familiarity, trust and flexibility, and sometimes caring responsibilities were shared across different family members [40, 44, 49, 54].

*I recognise that because my parents are from the Asian subcontinent*,* they have certain needs which actually my partner fulfils better than any paid service or home carer*,* anybody else would do. (Male carer*,* son*,* UK* [44]*)*

However, informal support networks were not available to everyone and were impacted by stigma, highlighting that HSCPs must not assume its presence [40, 44, 47, 48, 51, 53, 58, 61].

### Interpersonal level

#### Language barriers

Across all papers from countries where South Asians form a minority group, language barriers were a major obstacle to care planning (including ACP), decision-making, post-diagnostic support and quality of care. Family carers described difficulties with HSCPs not adequately comprehending different South Asian languages/dialects, leading to incorrect assumptions when providing care workers:

*And first of all*,* a lot of care agencies did not understand that there is a difference between – which is just stupid – that there is a difference between Punjabi*,* Gujarati and Hindi*,* like you cannot just send me a Gujarati speaking care worker and say that they understand Punjabi because it’s not the same. (Female carer*,* daughter*,* UK* [52]*)*

Language barriers negatively impacted the standard of care received by South Asian people with dementia in terms of interactions, assessments, information provision and treatment options [40, 42, 49–52]:

*Language barriers may prevent residents from participating successfully when they are treated by allied health staff*,* such as physios. (Professional – Care home worker*,* Australia* [42]*)*

Interpreters - formal, bilingual colleagues or family - helped communication, but were hindered by time constraints, difficulties doing virtual consultations and challenges around discussing sensitive topics and negotiating competing agendas [49, 50]. Jointly delivered training of interpreters together with HSCPs was mentioned as one way of addressing these difficulties.

*Sometimes you don’t get the full picture of what’s going on*,* because they [interpreters] say what they want to say and what you want to hear and not what you’re meant to be hearing. (Professional*,* community nurse*,* UK* [50]*)*

Other recommendations included translated resources, utilising bilingual staff, 24-hour multilingual helplines, language cards, recruiting more South Asian staff and providing existing staff with South Asian language training [42, 47, 50, 52].

#### Person-centred and family-centred care

Across most papers, adopting a person-centred approach was a key facilitator to building trust with South Asian people with dementia and family carers, addressing communication challenges, and encouraging holistic, empathetic support, which helped improve the acceptability and appropriateness of care offered to families:

*…even when I trained the focus was on the individual and trying to move away from labels and trying to make care more personal. So*,* for each new person that stands in front of me*,* I need to think about their experience*,* and where they are in the world and*,* how their…what’s happening to them is affecting them and those that love them… (Professional – community nurse*,* UK* [50]*)*

However, person-centred approaches varied between countries where South Asians form a majority population and countries where they represent a minority group. Across both settings, role reversal between parents and children was perceived, to some degree, as a natural consequence of dementia and linked to ideas of filial duty, love and *seva* (the cultural tradition of caring for elders in India [59]), . However, in India and Pakistan, this view was more pronounced and was also evident in HCPs’ attitudes, with greater infantilisation of people with dementia [59, 60]:

*We have to treat*,* and we have to take care of [a person with dementia] like a small kid. (Professional - nurse*,* India* [59]*)*

A barrier to person-centred care, in countries where South Asians represent a minority population, was a lack of integrated transcultural care training for HSCPs [42, 44, 45, 50]. However, in one study (Jenkins & Kamal, 2024), nurses noted that seniority in their roles and awareness of unconscious biases and cultural traditions, learned through South Asian colleagues or extra training modules, helped them offer better person-centred care:

*When you have an awareness of that sort of thing [referring to the importance of removing shoes indoors]*,* you can talk about it before anybody even has to ask you and then that helps you to build up a rapport*,* but I only know that through experience. If a new*,* qualified nurse came in with no experience they wouldn’t know that*,* there’s no training on that*,* you just learn as you’re going. (Professional – community nurse*,* UK* [50]*)*

Some papers [42, 50, 52, 53] suggested that HSCPs and care homes could also learn from family members, utilising them as ‘cultural brokers’ to share culturally relevant information (e.g., teaching staff keywords in the correct dialect and highlighting specific religious, dietary and personal care needs):

*I don’t know a lot about dementia and from what I can see*,* services mainly cater for white English people so if someone could come and educate me about dementia*,* I can educate them about the things that are important to my Mum*,* like her culture and religion. (Male carer*,* son*,* UK* [52]*)*

Family-centred approaches were valued across several papers [38, 40, 42, 50, 54], with poor communication with family carers identified as a major barrier to effective care planning and decision-making:

*The actual medical support she got was very thorough. I couldn’t fault that. But the problem was they were telling her things. She didn’t have a clue what was happening. So*,* no one communicated to me what was going on (Female carer*,* daughter*,* UK* [38]*)*

Professionals who established relationships and worked together with families were able to provide successful care planning and decision-making support:

*We built a relationship with*,* not just with the service user*,* but with nearly the entire family …And I got to know all of them*,* just because I was caring for their family*,* and it went really well. (Professional – in-patient nurse*,* UK* [50]*)*

### Organisational and systems level

#### Lack of integrated support

Across all papers, from both higher- and lower-income countries, there was a lack of integrated post-diagnostic support for South Asian people with dementia and family carers; this included limited information and support around diagnosis and prognosis, lack of signposting to appropriate services, no proactive ACP, and no clear post-diagnostic support pathways. Family carers often came across services by accident, had to do their own research. or were overwhelmed by too much information [52, 57].

*…and it was just really a case of we saw a consultant who said that there’s no cure for dementia*,* Alzheimer’s and dementia and this is your lot basically you’ve just gotta cope. There wasn’t any sort of support mechanism. (Carer*,* UK* [46]*)*

Although GPs were typically the clear first point of contact for diagnosis in higher-income countries, onward referral delays, bureaucracy, uncertainties over clinical responsibilities and lack of follow-up left many South Asian people with dementia and family carers feeling frustrated [40, 43, 47, 52, 53]:

*GP referred to someone else*,* a girl came from Middlemore about 5–6 months before and asked about memory loss problem….like you people are asking about…and did not contact again…(Person with dementia*,* New Zealand* [53]*)*

This frustration was also felt by HSCPs due to uncertainty and lack of training about which services to signpost to:

*I think the Trust just expect you to know where to signpost to*,* it’s just expected that you know it really. (Professional – community nurse*,* UK* [50]*)*

One recommendation from a UK-based paper included creating a standardised pathway, with resources available in different languages:

*A digital system so they could interact in languages […] a chart of what the process is if you think someone’s got dementia and who to go to and what service and what the process [is] to finding it out. And then where to go from there and what are those. (Female carer*,* daughter*,* UK* [52]*)*

In lower-income countries (India and Pakistan), barriers were more related to a lack of specialist dementia services, especially in rural areas, and concerns around dementia competency amongst HCPs, which resulted in misdiagnoses, conflicting medical advice or an overreliance on medication [41, 60, 61]:

*They are misdiagnosed and treated for something else*,* they are treated for mental disorders*,* they are treated for OCD*,* obsessive-compulsive disorder*,* for that they are given medicines. (Healthcare professional*,* India* [60]*)*

### Cultural competence

Across all papers from countries where South Asians represent a minority population, family carers and people with dementia reported that care planning and post-diagnostic support services did not meet their cultural needs. Cultural needs varied by subculture and religion, but centred around dietary preferences, personal care, language needs, and preferences for same-sex or culturally similar care workers:

*Here [in the UK] are just not tailored to meet the needs of our people. But that’s what you get in a different country. India was one thing*,* East Africa was something else and here.well….it’s this. (Female carer*,* daughter-in-law*,* UK* [51]*)*

Culturally appropriate personal care included using water to wash after toileting [40, 45–47, 49], highlighting the need for washroom facilities in day centres. In home settings, washing required more time, but families struggled to access the extra care worker hours needed, leading some to manage personal care themselves (although for others this was deemed culturally inappropriate), or spend time supervising or training care workers who were unfamiliar with this practice.

*It was not appropriate at all. Then I had to ask how they should do it. And eventually they did it. Because they used to use only wipers to wipe. They didn’t use water. But if you use water after [the] wipe*,* that cleans much better. They need to understand each culture. If you want to work with other people you need to know their culture. That’s why I have been doing this for mom. (Male carer*,* son*,* UK* [47]*)*

Linked to preferences around personal care, South Asian people with dementia often preferred same-sex care workers or those from similar religious or cultural backgrounds, who understood their cultural needs and spoke the same language, although priorities around this varied [40, 42, 44, 46, 47, 49, 50]:

*When the social worker was arranging the care*,* I just said mum is only going to agree to female carers. I know some people like people from their community*,* but my mum’s ok with anyone as long as they’re female. (Female carer*,* daughter*,* UK* [49]*)*

Across several papers [39, 42–44, 47, 49, 50, 56], there were concerns that care workers, day centres and care homes would not be able to provide culturally or religiously appropriate foods (e.g. halal or vegetarian meals):

*I think it’s quite difficult to go to a completely different place and have completely different food*,* in terms of if you’re Asian and then just English food. I think it’s important that they have things that they’re used to. (Person with dementia*,* female*,* UK* [56]*)*

Two papers [42, 56] highlighted the importance of culturally familiar foods in supporting nutrition, with recommendations for South Asian meal delivery services in care homes and culturally-tailored nutritional advice for managing eating and drinking difficulties.

One UK paper [46] found that South Asian families lacked support and faced barriers to culturally appropriate care even after death. Repatriating bodies back to home countries for funerals, sometimes at huge financial and emotional cost, went frequently unrecognised by mainstream services. Furthermore, delays in signing death certificates or releasing bodies (e.g., after post-mortems) impacted funeral arrangements, particularly for those from Muslim backgrounds, who require quick burials.

*Religious thing as well because when it’s time to go*,* it’s time to go. When somebody’s buried*,* it kind of eases the pain when they’ve gone. But if they’re hanging around in the mortuary or you know*,* in a freezer somewhere*,* you’ve always got that kind of like pain that they’re still*,* they haven’t gone yet. (Carer*,* UK* [46]*)*

South Asian-specific services (both mainstream and voluntary sectors – including South Asian-led day centres, carer support groups, dementia hubs (which offer activities, advice and multidisciplinary support for people with dementia), sitting services, outreach projects and faith-based initiatives) were viewed in several papers as being better able to meet cultural needs [39, 40, 42, 44, 47, 49, 52, 57]. They were felt to be more inclusive, had greater South Asian representation and elicited better trust and engagement from South Asian communities.

*There is a dementia group*,* not where we live*,* but where our daughter lives*,* and my daughter makes sure she takes both of us to the group [.]. Because in that group they have Indian songs*,* Indian food*,* and exercise. (Male carer*,* husband*,* UK* [49]*)*

However, differences within South Asian subcultures meant that such services were often not inclusive to all [42, 49, 51, 52]:

*She spoke Bengali*,* but she was a Hindu*,* she wasn’t a Muslim so she couldn’t take part in [the Bengali day centre]. Because she was vegetarian*,* and their prayer routine is different from hers*,* she just felt that she had nothing in common with them. (Professional – nurse*,* UK* [49]*)*

Two UK papers [39, 40] emphasised that tensions sometimes existed between voluntary and mainstream services regarding funding, referral pathways and responsibilities. Voluntary services were often overwhelmed by local need to fill the gap in mainstream services but felt a lack of recognition and reported a need for better dementia competency training. Some HSCPs argued for a flexible mainstream dementia service model that met the needs of the South Asian community rather than specialist South Asian services:

*I can’t see how the service can set up dozens and dozens of different models to meet the needs of different subgroups of the community. My answer is*,* you have a single service. It must be flexible and adaptable to meet the needs of different groups. (Professional*,* UK* [40]*)*

Other recommendations for enhancing cultural competency, particularly in day centres and care homes, included offering culturally meaningful activities, such as cultural festivals, movies, games and music, as well as environments that catered for cultural sensitivities around gender and religion [42, 49, 52, 53]:

*Besides I am thinking that there should be a community here where there should be activities like playing carrom [popular board game in India]. He would get better in this way*,* he would talk or play or look around*,* there was nothing like this what I would wish to. Not like*,* you go there and sit*,* then someone would feed you on time*,* just keep watching tv. You can watch that while staying at home. (Carer*,* New Zealand* [53]*)*

Several papers also emphasised the need to work in partnership with South Asian community organisations to co-produce culturally competent services and resources [42, 46, 50, 52, 53, 56, 57]:

*Aged care facilities do not have to be experts in everything and should collaborate with Indian community organisations to provide culturally appropriate care to their residents. (Professional – Care home worker*,* Australia* [42]*)*

Further recommendations included improved transcultural staff training, guidance on formulating culturally appropriate care plans and creating a directory with key information about South Asian cultures and religions [40, 42, 47, 50].

#### System pressures

Across most papers (from both higher- and lower-income countries), system pressures were a significant barrier to care planning and post-diagnostic support provision. Issues included time constraints, poor continuity of care, difficulties accessing GP/doctor appointments, long service waiting times, reduced funding and capacity, limited service choice, lack of transport, location-dependent service availability and restrictive eligibility criteria (e.g., for additional care worker hours):

*After some time they got in touch with me and they said we’ve observed that you are in the house with them in the evening*,* we are under the cosh*,* budgets*,* unfortunately we cannot send someone in the evening*,* can you manage that yourself? (Male carer*,* son*,* UK* [44]*)*

In countries where South Asians make up a minority population, system pressures also impacted the ability of services to meet cultural needs, such as being able to offer same-sex care workers, deliver tailored care or fund culturally specific services and training:

*At present*,* the very low numbers of residents with an Indian heritage means that it is not cost effective to provide culturally specific services… facilities cater for the majority residents they house. (Carer*,* Australia* [42]*)*

Though day centres were valued in both higher- and lower-income countries, there were often problems with transport, accessibility for those with mobility issues or advanced dementia, and lack of locally available options [49, 50, 52–54, 58]:

*The other problem is that there’s only one South Asian day centre. It’s completely the opposite end of the borough to most of our Asian populations. So they’re going to be on a bus for well over an hour each way and for some of them*,* that’s just too much. (Professional – clinical psychologist*,* UK* [49]*)*

In lower-income countries, lack of social welfare funding meant that most families paid privately for home care workers. In higher-income countries with established welfare systems, difficulties around accessing adequate or culturally-appropriate home care workers meant that many South Asian families also ended up paying privately, which could be costly [47, 49, 51–53], or considered relocating relatives back to their home countries for more affordable, culturally appropriate care [41, 56].

*Moreover*,* for cultural reasons my wife cannot look after him alone and cannot take him to the toilet which is not appropriate. That’s why I have to pay someone to look after my father. […] The caring agency was giving their maximum support but that was not enough for me. And I cannot ask for more as they can’t give so I paid for a carer. (Male carer*,* son*,* UK* [47]*)*

#### Institutional racism and discrimination

Across several UK papers [38, 40, 43, 45, 47, 51, 52], South Asian participants expressed fears around institutional racism and discrimination, leading to reduced trust in health and social care services. Older South Asian migrants who came to the UK in the 1960s–70s recalled racism influencing subsequent feelings of inclusion, while newer migrants showed greater acceptance of unequal treatment, affecting perceptions of entitlement to support:

*Even though we have a better standard of living in the UK*,* we are always going to be different here. It has been like that since we arrived you know…so you have to be persistent with everything. (Female carer*,* daughter-in-law*,* UK* [51]*)*

Some South Asian family carers feared discrimination during healthcare interactions, or perceived inequality in access to support services as being due to their ethnicity:

*Social carers getting money for providing care for my mother but when they come to my house they asked me to do all the work for them. They are not doing their jobs properly*,* they are not treating us equally as white people. (Female carer*,* daughter*,* UK* [45]*)*

Discrimination also took the form of institutions and HSCPs stereotyping South Asian people; for example, making assumptions about language ability, relying on South Asian families to provide care without additional resources, or viewing South Asian family carers as financially motivated [38, 45, 47, 49–51]:

*And then he says*,* does he speak English? At that point I said to him*,* should I tell you something? I’ll tell you something. He has been in this country since (19)59. He was a bank manager all his life. I was a head teacher. And now I leave it to you to decide whether we speak English or not (Female carer*,* wife*,* UK* [38]*)*

Facilitators to address institutional racism included better transcultural staff training, recruitment of more South Asian HSCPs and dedicated equality and diversity organisational leads [42, 47, 50, 52, 56].

### Structural level

#### Socioeconomic and policy factors

Broader structural factors also shaped the context within which care-planning and post-diagnostic support took place. In higher-income countries, changing family structures, due to occupational mobility and more South Asian women being in employment, meant that attitudes and capacity to take on traditional caregiving roles were shifting, highlighting the need for service adaptation [40, 44, 49, 56]. In both higher- and lower-income countries, financial constraints limited access to privately-funded care workers, transport to services, or investigations/treatments (particularly in countries with privately funded health and social care systems) [40, 49, 58, 61], whilst filling in gaps in care provision frequently negatively impacted family carers’ employment and income. Lower levels of education also contributed to some family carers feeling less confident when navigating services [51, 58, 61].

*A lot more South Asian families are working class and have issues with residency and access to benefits and pensions [….]. Whereas White British is a lot more middle-class*,* so they have easier access to paid services*,* which are flexible and easier to find*,* whereas for working class people who don’t have the money*,* it’s a lot more inflexible. (Professional – dementia support worker*,* UK* [49]*)*

In India, the 2007 Maintenance and Welfare of Parents and Senior Citizens Act, which places a statutory duty on adult children to support their parents, strongly influenced care expectations [59, 60]. A perceived lack of regulations in lower-income countries also influenced levels of trust, with greater fears of abuse of people with dementia by care workers, as well as fears of exploitation from private healthcare providers [60, 61].

*When we had all gone out*,* he (hired caretaker) had stuffed my father’s mouth with a cloth to stop him from repeating his words. (Carer*,* India* [61]*)*

## Discussion

The majority of papers reviewed (*n* = 20) reported on experiences and views from higher-income countries where South Asians represent a minority population. Papers mostly explored individual and community factors affecting acceptability and engagement with post-diagnostic support services (such as stigma, support networks and cultural duty to provide home care), as well as interpersonal, organisational and structural barriers affecting access to and provision of care (such as lack of culturally competent services, communication barriers, system pressures, fragmented support services, institutional discrimination and socioeconomic barriers). Only three papers specifically addressed care planning processes [42, 46, 50], but many others mentioned it within the context of social / home care (in relation to cultural and religious care needs and family involvement in care), suggesting care planning is under-researched and inconsistently applied, especially in primary care. Nonetheless, several influential factors, barriers and facilitators were identified, to help guide future care planning interventions for South Asian communities. Table 4 below summarises illustrative quotes for each theme according to its corresponding SEM level.

**Table 4:** Illustrative quotes linked to themes within each Socio-ecological model (SEM) level

Key influential factors	Supporting quotes
Individual and community level	
Cultural duty for home care	*But the South Asian community*,* when I consider my father-in-law*,* he always thinks that it’s his wife’s duty [to undertake all caring duties] and he didn’t need any carers [care workers] to come in and go out*,* it all has to be done by his wife.* (*Male carer*,* son-in-law*,* UK*,* S19* [56]) *We don’t need advice anymore. We need somebody to come and help us. We need somebody to come and give us some respite. We need money. We need funding. We don’t need talks. *(*Carer, UK, S3* [40]) *“Manush-e Kita Khoibo?” [what are people going to say? ]. How could we show our face to the community? It is not our culture to send older people to the care home. However*,* my children will also not feel good to send their mum to a care home.* (*Person with dementia*,* female*,* UK*,* S11* [48])
Stigma and misinformation	*I tell my friends and extended family members that I cannot remember things or forget recent events. However*,* I do not tell them that I have dementia; people will not understand what dementia is*,* and they may judge me. People are not educated. People might say-“e beti pagol oi gese” [she has gone mad]; then the whole community may gossip about my family and me*,* which may damage my family’s “izzat” [honour]*. (*Person with dementia*,* female*,* UK*,* S11* [48]) *… like social media that informs that what it is*,* what kind of disease it is. I have read and learnt a lot of things relating to the disease of my mother-in-law from the Internet. My husband has also read about it*.(*Female carer*,* daughter-in-law*,* Pakistan*,* S21* [58])
Support networks	*…it’s nice to see that they’ve all got a big family and it creates a positive impact on them I suppose*,* seeing familiar faces and making sure that their family are there every day and making sure their needs are met. Sometimes when they’re here we don’t really need to do anything for them…so*,* say when families do come they’ve actually said*,* oh no they like to bath them*,* they like to feed them*,* they like to take them to the toilet and they like to be involved in their care…* (* Professional – in-patient nurse*,* UK*,* S13* [50]) *I recognise that because my parents are from the Asian subcontinent*,* they have certain needs which actually my partner fulfils better than any paid service or home carer*,* anybody else would do*. (*Male carer*,* son*,* UK*,* S7* [44])
Interpersonal level	
Language barriers	*And first of all*,* a lot of care agencies did not understand that there is a difference between – which is just stupid – that there is a difference between Punjabi*,* Gujarati and Hindi*,* like you cannot just send me a Gujarati speaking care worker and say that they understand Punjabi because it’s not the same*. (*Female carer*,* daughter*,* UK*,* S15* [52]) *Language barriers may prevent residents from participating successfully when they are treated by allied health staff*,* such as physios*. (*Professional – Care home worker*,* Australia*,* S5* [42]) *Sometimes you don’t get the full picture of what’s going on*,* because they [interpreters] say what they want to say and what you want to hear and not what you’re meant to be hearing*. (*Professional – community nurse*,* UK*,* S13* [50])
Person-centred and family-centred care	*…even when I trained the focus was on the individual and trying to move away from labels and trying to make care more personal. So*,* for each new person that stands in front of me*,* I need to think about their experience*,* and where they are in the world and*,* how their…what’s happening to them is affecting them and those that love them…* (*Professional – community nurse*,* UK*,* S13* [50]) *We have to treat*,* and we have to take care of [a person with dementia] like a small kid*. (*Professional – nurse*,* India*,* S22* [59]) *When you have an awareness of that sort of thing [referring to the importance of removing shoes indoors]*,* you can talk about it before anybody even has to ask you and then that helps you to build up a rapport*,* but I only know that through experience. If a new*,* qualified nurse came in with no experience they wouldn’t know that*,* there’s no training on that*,* you just learn as you’re going*. (*Professional – community nurse*,* UK*,* S13* [50]) *I don’t know a lot about dementia and from what I can see*,* services mainly cater for white English people so if someone could come and educate me about dementia*,* I can educate them about the things that are important to my Mum*,* like her culture and religion*. (*Male carer*,* son*,* UK*,* S15* [52]) *The actual medical support she got was very thorough. I couldn’t fault that. But the problem was they were telling her things. She didn’t have a clue what was happening. So*,* no one communicated to me what was going on* (* Female carer*,* daughter*,* UK*,* S1* [38]) *We built a relationship with*,* not just with the service user*,* but with nearly the entire family …And I got to know all of them*,* just because I was caring for their family*,* and it went really well*. (* Professional – in-patient nurse*,* UK*,* S13* [50])
Organisational and systems level	
Lack of integrated support	*…and it was just really a case of we saw a consultant who said that there’s no cure for dementia*,* Alzheimer’s and dementia and this is your lot basically you’ve just gotta cope. There wasn’t any sort of support mechanism*. (*Carer*,* UK*,* S9* [46]) *GP referred to someone else*,* a girl came from Middlemore about 5–6 months before and asked about memory loss problem….like you people are asking about…and did not contact again…* (*Person with dementia*,* New Zealand*,* S16* [53]) *I think the Trust just expect you to know where to signpost to*,* it’s just expected that you know it really. (Professional – community nurse*,* S13*,* UK* [50]) *A digital system so they could interact in languages […] a chart of what the process is if you think someone’s got dementia and who to go to and what service and what the process [is] to finding it out. And then where to go from there and what are those.* (*Female carer*,* daughter*,* UK*,* S15* [52]) *They are misdiagnosed and treated for something else*,* they are treated for mental disorders*,* they are treated for OCD*,* obsessive-compulsive disorder*,* for that they are given medicines*. (*Healthcare professional*,* India*,* S23* [60])
Cultural competence	*Here [in the UK] are just not tailored to meet the needs of our people. But that’s what you get in a different country. India was one thing*,* East Africa was something else and here…well….it’s this*. (*Female carer*,* daughter-in-law*,* UK*,* S14* [51]) *It was not appropriate at all. Then I had to ask how they should do it. And eventually they did it. Because they used to use only wipers to wipe. They didn’t use water. But if you use water after [the] wipe*,* that cleans much better. They need to understand each culture. If you want to work with other people you need to know their culture. That’s why I have been doing this for mom*. (*Male carer*,* son*,* UK*,* S10* [47]) *When the social worker was arranging the care*,* I just said mum is only going to agree to female carers. I know some people like people from their community*,* but my mum’s ok with anyone as long as they’re female*. (*Female carer*,* daughter*,* UK*,* S12* [49]) *I think it’s quite difficult to go to a completely different place and have completely different food*,* in terms of if you’re Asian and then just English food. I think it’s important that they have things that they’re used to*. (*Person with dementia*,* female*,* UK*,* S19* [56]) *Religious thing as well because when it’s time to go*,* it’s time to go. When somebody’s buried*,* it kind of eases the pain when they’ve gone. But if they’re hanging around in the mortuary or you know*,* in a freezer somewhere*,* you’ve always got that kind of like pain that they’re still*,* they haven’t gone yet*. (*Carer*,* UK*,* S9* [46]) *There is a dementia group*,* not where we live*,* but where our daughter lives*,* and my daughter makes sure she takes both of us to the group […]. Because in that group they have Indian songs*,* Indian food*,* and exercise*. (*Male carer*,* husband*,* UK*,* S12* [49]) *She spoke Bengali*,* but she was a Hindu*,* she wasn’t a Muslim so she couldn’t take part in [the Bengali day centre]. Because she was vegetarian*,* and their prayer routine is different from hers*,* she just felt that she had nothing in common with them*. (*Professional - nurse*,* UK*,* S12* [49]) *I can’t see how the service can set up dozens and dozens of different models to meet the needs of different subgroups of the community. My answer is*,* you have a single service. It must be flexible and adaptable to meet the needs of different groups*. (*Professional*,* UK*,* S3* [40]) *Besides I am thinking that there should be a community here where there should be activities like playing carrom [popular board game in India]. He would get better in this way*,* he would talk or play or look around*,* there was nothing like this what I would wish to. Not like*,* you go there and sit*,* then someone would feed you on time*,* just keep watching tv. You can watch that while staying at home*. (*Carer*,* New Zealand*,* S16* [53]) *Aged care facilities do not have to be experts in everything and should collaborate with Indian community organisations to provide culturally appropriate care to their residents*. (*Professional – Care home worker*,* Australia*,* S5* [42])
System pressures	*After some time they got in touch with me and they said we’ve observed that you are in the house with them in the evening*,* we are under the cosh*,* budgets*,* unfortunately we cannot send someone in the evening*,* can you manage that yourself*? (*Male carer*,* son*,* UK*,* S7* [44]) *At present*,* the very low numbers of residents with an Indian heritage means that it is not cost effective to provide culturally specific services … facilities cater for the majority residents they house*. (*Carer*,* Australia*,* S5* [42]) *The other problem is that there’s only one South Asian day centre. It’s completely the opposite end of the borough to most of our Asian populations. So they’re going to be on a bus for well over an hour each way and for some of them*,* that’s just too much*. (*Professional – clinical psychologist*,* UK*,* S12* [49]) *Moreover*,* for cultural reasons my wife cannot look after him alone and cannot take him to the toilet which is not appropriate. That’s why I have to pay someone to look after my father. […] The caring agency was giving their maximum support but that was not enough for me. And I cannot ask for more as they can’t give so I paid for a carer*. (*Male carer*,* son*,* UK*,* S10* [47])
Institutional racism and discrimination	*Even though we have a better standard of living in the UK*,* we are always going to be different here. It has been like that since we arrived you know…so you have to be persistent with everything*. (*Female carer*,* daughter-in-law*,* UK*,* S14*[51]) *Social carers getting money for providing care for my mother but when they come to my house they asked me to do all the work for them. They are not doing their jobs properly*,* they are not treating us equally as white people*. (*Female carer*,* daughter*,* UK*,* S8* [45]) *And then he says*,* does he speak English? At that point I said to him*,* should I tell you something? I’ll tell you something. He has been in this country since (19)59. He was a bank manager all his life. I was a head teacher. And now I leave it to you to decide whether we speak English or not* (*Female carer*,* wife*,* UK*,* S1* [38])
Structural level	
Socioeconomic and policy factors	*A lot more South Asian families are working class and have issues with residency and access to benefits and pensions [….]. Whereas White British is a lot more middle-class*,* so they have easier access to paid services*,* which are flexible and easier to find*,* whereas for working class people who don’t have the money*,* it’s a lot more inflexible*. (*Professional – dementia support worker*,* UK*,* S12* [49]) *When we had all gone out*,* he (hired caretaker) had stuffed my father’s mouth with a cloth to stop him from repeating his words*. (*Carer*,* India*,* S24* [61])

*Not all studies supplied demographic information for quotes

**Fig. 4:**
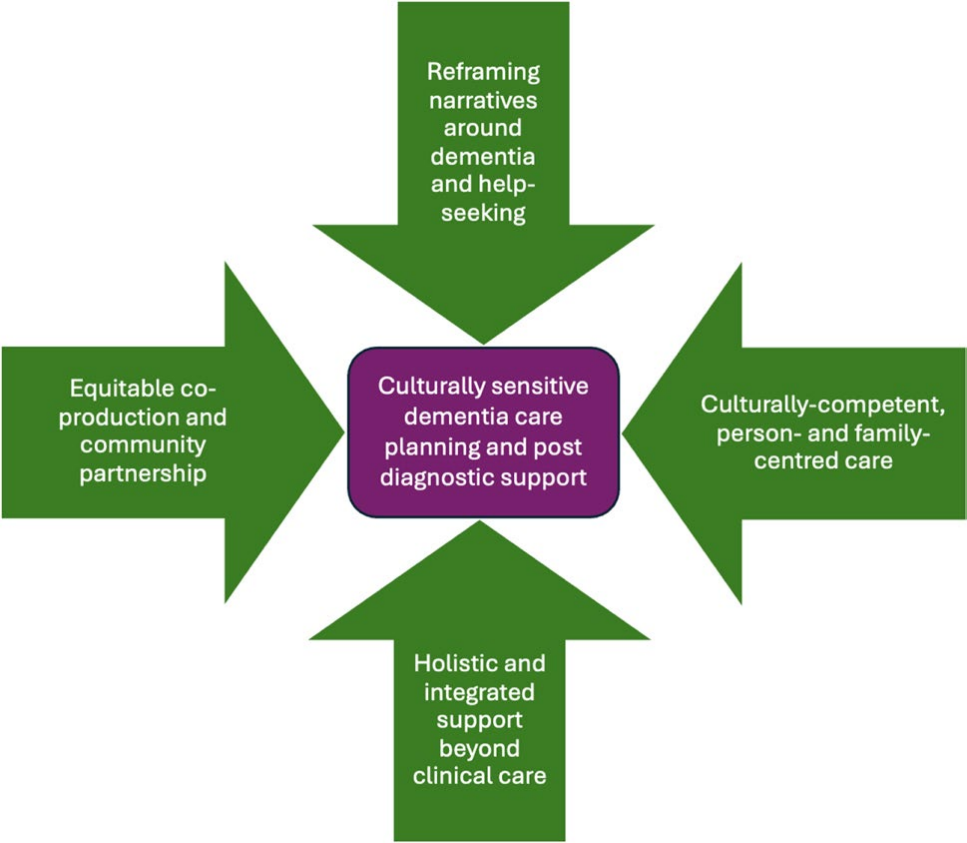
Recommendations to improve care planning and post-diagnostic support for South Asian people with dementia

We have arranged the themes across papers into four overarching recommendations to improve culturally-sensitive care planning and post-diagnostic support for South Asian people with dementia and family carers (mostly applicable to higher-income countries where South Asians represent a minority population), as illustrated in Fig. 4: (1) reframing narratives around dementia and help-seeking; (2) culturally competent, person- and family-centred care; (3) holistic and integrated support beyond clinical care; (4) equitable co-production and community partnership. Within the scope of each of these recommendations, we also compare findings from the four papers from India and Pakistan (where South Asians form the majority population).

### Reframing narratives around dementia and help-seeking

Whilst generational shifts in dementia awareness were noted, across all reviewed papers, stigma, misinformation and low dementia literacy remain major barriers to engagement with care planning and post-diagnostic support services in South Asian populations. This reflects and builds on existing studies which have identified these factors as major contributors to delayed dementia diagnosis in this group [4, 9, 16, 28]. There is evidence that negative perceptions around dementia hinder proactive care planning [21]. Our review supports the need to reframe dementia within South Asian communities – not just as a medical condition, but as something people can live well with [15] - in order to reduce stigma and normalise early help-seeking. In countries where South Asians make up a minority group, culturally-tailored public health messaging and local education campaigns were recommended, enacted through workshops, South Asian media outlets (e.g. local radio, TV, talk-shows and social media) and religious institutions (such as Gurdwaras and Mosques). In India and Pakistan, *national* awareness campaigns about dementia using a variety of media outlets, but fewer religious channels [58], were favoured.

### Culturally competent, person- and family-centred care models

All papers from countries where South Asians represent a minority population expressed the need for culturally attuned, person-centred dementia care planning and post-diagnostic support. This echoes findings from a report by Jutlla [15], which highlighted White-centric services as being a greater barrier to engagement than stigma. A key finding from our review is the propensity for services to stereotype South Asian subcultures and dialects. Person-centred care has long been advocated as the gold standard for dementia care [62, 63], but our review highlights the need for a more culturally responsive approach, incorporating an intersectionality framework that takes into account societal and structural barriers [64]. Recommendations to improve cultural competency include the provision of translated information, culturally meaningful activities, adequately trained interpreters, guidance on formulating culturally tailored care plans and transcultural staff training, as well the ability to be responsive to South Asian dietary, personal care and religious needs. Recommendations for culturally appropriate ACP stressed supporting families to initiate discussions and consider cultural needs after death. Family-centred and collaborative care, underpinned by a sense of mutual knowledge and learning exchange between HSCPs and families, was also seen as crucial. South Asian families expressed a need for more involvement in dementia care planning and decision-making, although limits around autonomy and confidentiality need to be taken into consideration [65].

In contrast to cultural competency, most papers from India and Pakistan highlighted a need for improved dementia competency and training amongst HSCPs, and more specialist dementia services nationally. This review also highlights how concepts of person- and family-centred care vary across cultural and national contexts, suggesting the need for further research in this area.

### Holistic and integrated support beyond clinical care

This review underscores the need for flexible, accessible, community-anchored services to support *home-based care models*, including increased care worker hours, respite care, day centres, sitting services, carer education and psychosocial support for both family carers and people with dementia. This was noted across papers from both higher- and lower-income countries. Family carers also require practical assistance with advocacy, legal matters and accessing financial benefits, highlighting the need to broaden the scope of care planning beyond clinical care. These findings are not specific to South Asian populations and are in keeping with results from a recently co-produced paper exploring post-diagnostic support for people with dementia in general [66]. Dementia care planning for South Asian families should adopt a multidisciplinary approach with better integration between health, social care and community organisations. It would also be important to take multimorbidity and frailty into account when undertaking care planning, highlighting the need for comprehensive geriatric assessments, to ensure dementia is not considered in isolation, but in the context of other age-related conditions [67]. However, multidisciplinary care planning in the general population remains limited [21], with fragmented services, pressures on primary care and insufficient staff training. Given that service information is often inaccessible or overwhelming, there is a potential role for a care navigator or South Asian link worker [15, 68] to improve signposting, build trust and connect people to culturally competent community resources that address wider non-clinical needs. However, the current evidence base for these roles is limited [69, 70], highlighting the need for further evaluation.

### Equitable co-production and community partnership

Many participants in this review expressed distrust in institutions and HSCPs. In countries where South Asians are a minority population - to increase trust and create equitable and culturally competent dementia services, several papers recommended working together in partnership with South Asian communities and stakeholders, including community organisations, religious channels, trusted peer educators, local leaders and people with experiential knowledge of dementia, through the formation of ‘cultural hubs’ [42] - to design, commission and co-produce services and staff training. This would help tackle stigma, ensure services meet nuanced cultural needs (e.g., taking South Asian subcultures and dialects into account) and boost service uptake. Examples of successful co-production projects include the development of culturally-adapted dementia diagnostic assessment tools [71], the ADAPT toolkit [72], ACP workshops with South Asian older adults [73] and a culturally-adapted psychosocial intervention for family carers [74]. However, our review also underscores some inherent issues with partnership working between mainstream statutory services and voluntary, community, faith and social enterprises (VCFSEs), such as unequal power-sharing and resource distribution. Cheston et al. [75] additionally highlight a general reluctance to address services shortcomings within mainstream services due to unconscious biases reinforcing cultural stereotypes.

In papers from India and Pakistan, main recommendations to improve trust in dementia care services centred around better regulation of home care workers, potentially through local policy framework changes, and improved dementia training and education for HSCPs.

## Strengths and limitations

We systemically identified studies spanning diverse health and social care systems, South Asian subcultures, religions and socioeconomic contexts. However, our findings primarily apply to high-income Western countries, where South Asians represent a minority group, and not all South Asian subcultures are represented in this review. Whilst we have highlighted findings from countries where South Asians make up a majority population, the low number of papers (n-=4) make it difficult to draw firm or meaningful conclusions. Key strengths of this review include the comprehensive search / screening strategy, minimising the risk of missing relevant studies. Furthermore, a third of included studies explored the views and experiences of South Asian people with dementia, who are an underrepresented group in research.

However, papers varied in quality and, in studies reporting undifferentiated views in mixed populations, only clearly attributable data were analysed, potentially excluding relevant insights. The exclusion of studies exploring the views of paid carers (in the search strategy) is another limitation, particularly as findings highlight the important role of care workers in post-diagnostic support and care planning. However, no studies were in fact excluded based on this criterion, suggesting that care workers are a potentially under-represented group in research and that future studies should focus on incorporating their views and experiences. Although this review focused on dementia post-diagnostic support and care planning, most studies addressed the former, with none detailing specific care planning pathways or service development processes. This reflects broader inconsistencies in how care planning is delivered and reported, alongside an absence of standardised post-diagnostic dementia pathways. Furthermore, most studies in the review gave no indication of dementia severity, so we cannot comment on how this might affect engagement with and access to post-diagnostic support services and care planning.

Whilst a key strength of this review is its use of the adapted SEM [37] to illustrate how multilevel sociocultural factors influence care planning and post-diagnostic support, as well as identify possible intervention points – this model is perhaps weaker at identifying how overlapping identities (such as race, gender, class) come together to forge unique experiences of power and care (in contrast to intersectionality models [64]). Another key strength of this review is the involvement of a Patient and Public involvement (PPI) group, comprised of four South Asian current and former family carers of people with dementia, who set the priorities for the review questions and will help to formulate dissemination outputs. However, they were not actively involved in the analysis stage, which is a limitation.

## Conclusions

Our review highlights a pressing need to rethink how organisations and health and social care systems design and deliver dementia care planning and post-diagnostic support interventions for South Asian populations. In countries where South Asians are a minority population, instead of top-down approaches, co-production of services and reciprocal knowledge exchange between staff, families, people with dementia and communities are essential to address the cultural biases and stereotypes often embedded in current service design and care practices. This approach would help to commission services that are most relevant to South Asian families, reduce stigma and make knowledge about dementia more widely accessible. Future research should explore how best to incorporate co-production steps in service design, with the input of PPI. Only four papers explored the experiences of those in lower-income countries, where South Asians form a majority population, which suggests that further research is needed in these contexts; however, some priorities that emerged from these papers include strengthening dementia competency amongst HSCPs, expanding specialist dementia service provision and increasing public awareness nationally.

## Supplementary Information

Additional file 1. Appendix 1: Medline search terms for systematic review.

Additional file 2. Appendix 2: Data extraction form for systematic review.

Additional file 3. Appendix 3: PRISMA 2020 Checklist for reporting systematic reviews.

## Data Availability

The datasets used and/or analysed during the current study are available from the corresponding author on reasonable request. The study protocol has been registered on PROPSERO [Registration number: CRD42023404125]. Clinical trial number: not applicable.

## Abbreviations

ACP: Advance care planning
GP: General Practitioner
HCP: Healthcare professional
HSCP: Health and social care professionals
ME: Minority ethnic
PPI: Patient and Public involvement
SEM: Socioecological model
UK: United Kingdom
VCFSEs: Voluntary, community, faith and social enterprises
