# Supplementary material for: Key factors influencing post-diagnostic support and care planning for people with dementia from South Asian backgrounds: a systematic review of qualitative studies
Source: BMC Geriatr. 2026 Jan 31;26:205. doi: 10.1186/s12877-026-07064-y (PMC12895770; doi:10.1186/s12877-026-07064-y)
Supplement: Supplementary file 1 — Additional file 1. Appendix 1: Medline search terms for systematic review. [file 12877_2026_7064_MOESM1_ESM.docx]

## Appendix 1 (Additional File 1): Medline search terms for systematic review

| **Concept** | **MeSH terms** | **Keywords** |
| --- | --- | --- |
| Dementia | Dementia | “Alzheimer* disease” or “creutzfeldt-jakob disease” or “delirium” or “dementia” or “dementia diagnos*” or “dementia with lewy bodies” or “early onset dementia” or “fronto-temporal dementia” or “cognitive* impair*” or “huntington* disease” or “korsakoff* syndrome” or “memory loss” or “parkinson* disease” or “vascular dementia” |
| South Asian Ethnicity | Bangladesh  Or  India  Or  Pakistan  Or  Sri Lanka  Or  Nepal  Or  Bhutan  Or  “Ethnic and Racial Minorities” | “India*” or “Bangladesh*” or “Nepal*” or “Pakistan*” or “South Asia*” or “BME” or “BAME”^[[1]](#footnote-1)^ or “ethnic minorit*” or “muslim*” or “hindu*” or “Islam*” or “sikh*” |
| Qualitative Methods | Qualitative Research  Or  Focus Groups  Or  Interview  Or  Observational Study  Or  Exp Anthropology  Or  Interviews as topic  Or  Narration | “qualitative*” or “interview*” or “focus group*” or “observation*” or “audio” or “video” or “ethnograph*” or “fieldwork” or “field work” or “key informant*” or “conversation analysis” or “interpretat* phenomenologic* analys*” or “content analys*” or “framework analys*” or “thematic analy*s” or  “((“semi-structured” or “semistructured” or unstructured or informal or "in-depth" or indepth or "face-to-face" or structured or guide) adj3 (interview* or discussion* or questionnaire*)) |

1. Whilst no longer favoured in usage, BAME and BME terms were used in the search strategy to capture older papers that historically used this terminology. [↑](#footnote-ref-1)
