# Supplementary material for: Key factors influencing post-diagnostic support and care planning for people with dementia from South Asian backgrounds: a systematic review of qualitative studies
Source: BMC Geriatr. 2026 Jan 31;26:205. doi: 10.1186/s12877-026-07064-y (PMC12895770; doi:10.1186/s12877-026-07064-y)
Supplement: Supplementary file 2 — Additional file 2. Appendix 2: Data extraction form for systematic review. [file 12877_2026_7064_MOESM2_ESM.docx]

## Appendix 2 (Additional File 2): Data extraction form for systematic review

**DATA EXTRACTION FORM**

**Name of Study:**

**Author(s):**

Year:

**Publication:**

| **Heading** | **Subheading** | **For completion by reviewer** |
| --- | --- | --- |
| **Study Characteristics** | Bibliographic details |  |
|  | Reviewer name: | Reviewer 1  Reviewer 2  Reviewer 3 |
|  | Eligible? | Yes  No  Unclear |
|  | Reviewer’s subjective rating |  |
|  | Type of study |  |
|  | Participants | PWD Carers. Professionals:  Ethnicity:  Gender:  Age: |
|  | Study aims and purpose  Aims clearly stated? | Yes No Unclear |
|  | Key findings |  |
| **Setting** | Geographical and care setting for the study | Country:  Majority or minority experience?  Urban Rural Semi-Urban Semi-rural Mixed  Setting: Home Day Centre Hospital Nursing Home Care Home Community Other: |
|  | Sufficient detail about the setting? | Yes No Unclear |
|  | Date collection period |  |
| **Sample** | Inclusion Criteria |  |
|  | Exclusion Criteria |  |
|  | How was the sample selected? |  |
|  | Size of Sample |  |
|  | Appropriateness | Yes No Unclear |
| **Data Collection** | Methods | Interview: Observation Focus Group Mixed Methods Other: |
|  | Data analysis | Type of data analysis:  Strengths/weaknesses of analysis:  Validity/reliability of data? Any tools used: |
|  | Reflexivity | Yes No Unclear |
| **Outcomes** | Outcomes & Impact |  |
|  | Aspect of care planning or post-diagnostic support | - Provision of information/signposting to services - Health/wellbeing reviews - Care plan - interactions with professionals - Services and access to support - Care environment - Cultural factors - ACP or EOL focus - Facilitators/barriers |
| **Findings** | Themes |  |
|  | Conclusions |  |
| **Policy & Practice** | Generalisability? |  |
|  | Implications for policy |  |
|  | Implications for practice |  |
| **Other** | Forward citation tracking |  |
| **Decision** | *Should this study be included?* | Yes No Maybe |
|  | Agreement with reviewer? | Yes No |
